# Antioxidant and Anti-Inflammatory Properties of *Nigella sativa* Oil in Human Pre-Adipocytes

**DOI:** 10.3390/antiox8020051

**Published:** 2019-02-25

**Authors:** Laura Bordoni, Donatella Fedeli, Cinzia Nasuti, Filippo Maggi, Fabrizio Papa, Martin Wabitsch, Raffaele De Caterina, Rosita Gabbianelli

**Affiliations:** 1 Unit of Molecular Biology, School of Pharmacy, University of Camerino, 62032 Camerino MC, Italy; laura.bordoni@unicam.it (L.B.); donatella.fedeli@unicam.it (D.F.); 2 Unit of Pharmacology, School of Pharmacy, University of Camerino, 62032 Camerino MC, Italy; cinzia.nasuti@unicam.it; 3 Pharmaceutical Botany and Pharmacognosy Unit, School of Pharmacy, University of Camerino, 62032 Camerino MC, Italy; filippo.maggi@unicam.it; 4 School of Science and Technology, University of Camerino, 62032 Camerino MC, Italy; fabrizio.papa@unicam.it; 5 Division of Pediatric Endocrinology and Diabetes, Department of Pediatrics and Adolescent Medicine, Ulm University Medical Center, 89081 Ulm, Germany; Martin.Wabitsch@uniklinik-ulm.de; 6 Division of Cardiovascular Medicine, Cardio-Thoracic and Vascular Department, University of Pisa, 56126 Pisa, Italy; raffaele.decaterina@unipi.it

**Keywords:** *Nigella sativa*, antioxidant properties, inflammation, cytokines, human pre-adipocytes

## Abstract

The oil obtained from the seeds of *Nigella sativa* L. (*N. sativa*), also known as black cumin, is frequently used in the Mediterranean area for its anti-inflammatory, anti-oxidant, and anti-cancer activities. The aim of the present study was to evaluate the antioxidant and anti-inflammatory properties of the oil extracted from seeds of a *N. sativa* cultivar produced in the Marche region of Italy, and to determine if the thymoquinone content, antioxidant properties, and biological activity would decay during storage. Cytotoxicity and anti-inflammatory properties of *N. sativa* oil were tested in an in vitro model of low-grade inflammation in Simpson–Golabi–Behmel syndrome human pre-adipocytes. The fresh extracted oil (FEO) contained 33% more thymoquinone than stored extracted oil (SEO), demonstrating that storage affects its overall quality. In addition, the thymoquinone content in the *N. sativa* oil from the Marche region cultivar was higher compared with other *N. sativa* oils produced in the Middle East and in other Mediterranean regions. Pro-inflammatory cytokines (e.g., Interleukin (IL)-1alpha, IL-1beta, IL-6) were differently modulated by fresh and stored extracts from *N. sativa* oils: FEO, containing more thymoquinone reduced IL-6 levels significantly, while SEO inhibited IL-1beta and had a higher antioxidant activity. Total antioxidant activity, reported as µM of Trolox, was 11.273 ± 0.935 and 6.103 ± 0.446 for SEO and FEO (*p* = 1.255 × 10^−7^), respectively, while mean values of 9.895 ± 0.817 (SEO) and 4.727 ± 0.324 (FEO) were obtained with the 2,2-diphenyl-1-picryl-hydrazyl-hydrate (DPPH) assay (*p* = 2.891 × 10^−14^). In conclusion, the oil capacity to counteract proinflammatory cytokine production does not strictly depend on the thymoquinone content, but also on other antioxidant components of the oil.

## 1. Introduction

*Nigella sativa* L. (*N. sativa*), also known as black cumin, is a plant grown in Mediterranean countries, South and Southwest Asia, and is known for its content of bioactive compounds (i.e., tocopherols, vitamin A and C, β-carotene, etc.) in the seed. Notably, the seed biological activity has been associated with its thymoquinone content. However, the *N. sativa* seed contains other compounds, such as fixed oil (22–38%), volatile oil (0.40–1.5%), proteins (21–31%), carbohydrates (25–40%), minerals (3.7–7%), vitamins (1–4%), saponins (0.013%), and alkaloids (0.01%), which can all contribute to its biological properties [1].

Previous studies have highlighted that *N. sativa* oil has anti-inflammatory, anti-oxidant, immunomodulatory, and anti-cancer activities [2,3]. Recently, other investigations have addressed the possibility that *N. sativa* oil improves glucose homeostasis and lipid profile [4,5]. Additionally, pharmacological studies have highlighted that *N. sativa* oil can have gastroprotective, hepatoprotective, antitussive, cardioprotective, and anti-hypertensive properties [1]. *N. sativa* oil inhibits histamine release from mast cells, suggesting its potential use in asthma [3]. Its use has also been suggested as an adjunct therapy to reduce the toxic effects of cis-platin [6,7]. However, 1000 mg/day of oil did not prove effective in the management of the multi-system chronic inflammatory Behcet’s disease. The favorable effects of *N. sativa* oil were first associated with its thymoquinone content, which can vary from 0.05 to 6.19 mg/mL according to the area of production and the extraction method [8,9]. Furthermore, its biological activity has been related to the control of the redox system through its free radical–scavenging activity and modulation of the endogenous antioxidant systems. Liver upregulation of catalase (CAT), glutathione peroxidase, and superoxide dismutase (SOD) has also been observed in rats [10]. Diabetic rats treated with *N. sativa* seed powder and thymoquinone feature low blood malondialdehyde and higher superoxide dismutase levels [11], while pre-treatment with 10 mg/kg thymoquinone in rats restores liver superoxide dismutase activity and lipid peroxidation modified by paraquat exposure [12]. Recently, thymoquinone pre-treatment was shown to protect human retinal pigment epithelial cells from hydrogen peroxide-induced oxidative stress by activation of the Nuclear factor erythroid 2-related factor 2 (Nrf2)/ Heme oxygenase-1 (HO-1) pathway [13] and to favorably influence an animal model of arthritis [14].

Since the composition of the oil can vary considerably according to environmental factors (e.g., soil composition and climatic condition) and from the procedure of extraction and conservation of the oil, the present study aimed at investigating how thymoquinone content, anti-inflammatory, and anti-oxidant properties vary with the storage of the oil. Moreover, a goal of this study was to evaluate if the anti-inflammatory properties of *N. sativa* oil, measured in an in vitro model of low-grade inflammation in Simpson–Golabi–Behmel syndrome (SGBS) human pre-adipocytes, depends on thymoquinone concentration or on the presence of other oil components.

## 2. Materials and Methods

### 2.1. Materials

All reagents were of analytic grade and purchased from Sigma Chemical Co. St. Louis (St. Louis, MO, USA) if not otherwise noted. Thymoquinone (synFEO), thymol (IS), catalase, xanthine oxidase, and superoxide dismutase were obtained from Sigma–Aldrich (Milan, Italy). *Nigella sativa* oil was produced by Tre Ponti Snr factory, Polverigi (AN), Italy. Human monocytic cell line THP-1 cells were purchased from “Istituto Profilattico Sperimentale della Lombardia e dell’Emilia Romagna Bruno Ubertini” (Brescia, Italy). SGBS cells were kindly provided by Martin Wabitsch and Raffaele de Caterina.

### 2.2. SEO and FEO Production

Stored extracted oil (SEO) and fresh extracted oil (FEO) were obtained from the same cultivar. Seeds were stored at a controlled temperature (14 °C) in a dark room. Oils were extracted by cold pressing seeds using a squeezing machine (Vero Energia Italia S.r.l., Ravenna, Italy), and filtered after 10 days to remove solid residual. SEO was extracted from fresh seed and maintained for 12 months in a box away from any heat or sunlight before the study. FEO was extracted just before the study from seeds of the same cultivar, stored as reported above.

### 2.3. Quantitative Determination of Thymoquinone in SEO and FEO

**Figure 1:**
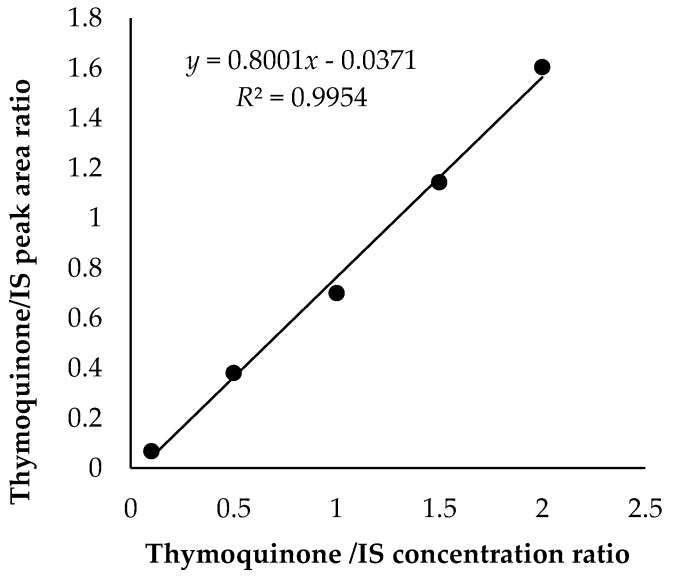
Calibration curve for quantitative determination of thymoquinone using thymol as internal standard (IS).

SEO and FEO were analysed for their content in thymoquinone by gas chromatography coupled to flame ionization detection (GC-FID) using thymol as internal standard (IS). For this purpose, a gas chromatograph Agilent 4890D, equipped with split/splitless injection port and FID detector (Agilent Technologies, Santa Clara, CA), was used. Separation of thymoquinone was achieved by using an apolar HP-5 capillary column (30 m × 0.25 µm i.d., 0.25 µm f.t., 5% phenylmethylpolysiloxane) from Agilent Technologies (Palo Alto, CA, USA). The temperature program of the oven was as follows: 2 min at 60 °C, subsequently 20 °C/min up to 280 °C, held for 7 min, for a total of 20 min. Injector and detector temperature were 280 °C. Helium (99.99%) was used as the carrier gas with a flow rate of 1.4 mL/min. The injection volume was 1 µL. Thymoquinone was diluted in *n*-hexane at various concentrations (0.01, 0.05, 0.1, 0.15, and 0.2 mg/mL keeping the concentration of IS at 0.1 mg/mL. These solutions were injected four times and peak areas of thymoquinone and IS were determined. For each analysis, the thymoquinone/IS peak area ratio was calculated and plotted versus the thymoquinone/IS concentration ratio (Figure 1). Afterwards, SEO and FEO were diluted 1:100 in *n*-hexane containing 0.1 mg/mL of IS and injected (1 µL) into GC-FID system in four replicates in three different days using the calibration method described above. Data were analysed by HP3398A GC Chemstation software (Hewlett–Packard, Rev. A.01.01 Santa Clara, CA).

### 2.4. Antioxidant Assays

The scavenger capacity of SEO and FEO was analyzed using different spectrophotometric assays (total antioxidant activity and 2,2-diphenyl-1-picryl-hydrazyl-hydrate (DPPH) assay and chemiluminescent tests (luminol-amplified- and lucigenin-amplified-chemiluminescence).

The total antioxidant activity (TAA) of different samples was obtained according to Pellegrini et.al. [15]. With this assay we measured the capacity of oils, synFEO or medium from SGBS incubation, to quench the blue/green chromophore of the 2,2’-azinobis-(3-ethylbenz-thiazoline-6-sulfonic acid) (ABTS) radical cation (diammonium salt), whose decrease in absorbance at 734 nm is proportional to the antioxidants present in the sample. The ABTS radical cation solution was prepared by dissolving 7 mM ABTS in water with 2.5 mM potassium sulphate and adding ethanol to reach an absorbance at 734 nm of 0.7 ± 0.2. 30 µL of FEO or SEO were mixed with 90 µL of hexane (1:3 dilution); 20 µL of this mixture was added to 2 mL of ABTS solution, incubated at room temperature in the dark for 10 min, and then spectrophotometric absorbance at 734 nm at 30 °C was determined. TAA was also determined in the SGBS pre-adipocytes growth medium after 24 h incubation with 1 µM synFEO, and with a volume of FEO corresponding to 1 µM synFEO and SEO at the same volume of FEO. 1 mL of medium from each sample was centrifuged and 20 µL were used in the assay, as already reported. The absorbances were referred to the calibration curve obtained in the presence of known concentrations of antioxidant Trolox (6-hydroxy-2,5,7,8-tetramethylchroman-2-carboxylic acid). Data are reported as concentration of Trolox (µM).

The antioxidant activity versus DPPH radical was determined following the method reported by Zullo and Ciafardini [16]. Briefly, DPPH test measures the capacity of antioxidants to scavenge the 2-diphenyl-1-picrylhydrazyl radical (DPPH) by spectrophotometrically measuring the decrease of absorbance of DPPH solution at 517 nm. 200 µL of oil was mixed with 600 microL of methanol, vortexed, centrifuged for 3 min, and then the upper methanolic phase was separated. 50 µL of this methanolic phase was added to 2 mL of 3 mM DPPH dissolved in methanol, incubated for 5 min at room temperature in a dark place and, finally, the absorbance at 517 nm was read in the spectrophotometer. The results were compared to the DPPH radical-scavenging activity of standard concentrations of Trolox and reported as concentration (µM) of Trolox.

Luminol and lucigenin-amplified chemiluminescence was used to detect the antioxidant activity of SEO and FEO to scavenge hydrogen peroxide and superoxide anion, respectively. Luminol is a chemiluminogenic probe that reacts with hydrogen peroxide to give a chemiluminescence signal. Similarly, lucigenin, which is sensitive to superoxide anion produced by the xanthine/xanthine oxidase system, reacts with superoxide anion to give a chemiluminescence signal proportional to the superoxide anion present in the medium [17,18]. In both assays, the chemiluminescence signal is reduced by the presence of the antioxidant. Luminol-amplified chemiluminescence was measured in 1 mL of 50 mM Tris buffer pH 7.4 with 0.1 mM luminol and 10 μL of SEO or FEO oil. The reaction was initiated by injecting hydrogen peroxide at the final concentration of 50 mM. Results were compared with the standard curve obtained in the presence of catalase and results are expressed as µg CAT/10 μL oil. Lucigenin-amplified chemiluminescence was measured in 1 mL of 50 mM Tris buffer pH 7.4 containing 0.1 U/mL xanthine oxidase, 150 μM lucigenin, and 10 µL of SEO or FEO oil. The reaction was started by injecting xanthine at a final concentration of 50 µM. Results were compared with the standard curve obtained in the presence of superoxide dismutase and results are expressed as microg SOD/10 µL oil. Chemiluminescence was measured in an Autolumat LB953 (Berthold Co. Wildbad, Germany).

In all antioxidant assays (TAA, DPPH and chemiluminescence assays), the chosen volume of compound was the minimum capable of giving an appreciable antioxidant effect in the analytical system.

### 2.5. Anti-Inflammatory Property

SGBS and monocytic leukemia (THP1) cell lines were cultured as previously described by Wabitsch and collaborators [19].

Cytotoxicity of *N. sativa* oil samples (FEO, SEO) and a solution equivalent to FEO in terms of synthetic thymoquinone concentration (synFEO) was tested by the 3-(4,5-dimethylthiazol-2-yl)-2,5-diphenyltetrazolium bromide (MTT) assay for 24 h (Fisher Scientific, Italia, Italy). Cells were plated in a 96-well plate at a seeding density of 1 × 10^4^ cells/well. After 24 h, the medium was replaced with 100 µL of medium containing FEO, SEO, or synFEO at different concentrations of thymoquinone (from 0.1 µM to 100 µM for synFEO and FEO and a volume for SEO as in FEO samples); samples were incubated for 24 h. At the end of the FEO treatment period, the medium was discarded and 50 µL of MTT (5 mg/mL in phosphate buffered saline) solution were added to each well. After 4 h of incubation at 37 °C, MTT was discarded, the formazan crystals were dissolved in 100 µL of dimethyl sulfoxide (DMSO) and absorbance was measured after 10 min using an ELISA reader (Fluostar Omega, BMG Labtech, Ortenberg, Germany) at 570 nm.

Inflammation was induced in SGBS pre-adipocytes by conditioning their culture medium with 15% of supernatant obtained after THP1 differentiation with phorbol 12-myristate 13-acetate (PMA) (Sigma–Aldrich, St. Louis, USA) for 48 h [19]. SGBS-inflamed cells were treated for 24 h with FEO, SEO, and synFEO, dissolved in DMSO. The volume of FEO was chosen to have a final content of thymoquinone of 1 microM. The same volume of SEO and FEO was used in cell cultures. The cytokine profile (Interleukin (IL)-1alpha, IL-1beta, IL2, IL4, IL6, IL8, IL10, IL12, IL17A, Interferon (IFN)-gamma, IFNγ, Tumor Necrosis factor (TNF) alpha, Granulocyte-macrophage colony-stimulating factor (GM-CSF) in the supernatant was assessed after 24 h using the Multi-Analyte ELISArray Kits (Qiagen, Venlo, Netherlands) according to the manufacturer’s instructions.

### 2.6. Statistical Analysis

The Shapiro–Wilk test was applied to test normality of data distribution. Paired and independent *t*-tests were used to identify significant differences for TAA, DPPH, and chemiluminescence assays (Shapiro–Wilk, *p* > 0.05). The Kruskal–Wallis and Mann–Whitney U tests were used to check for statistically significant differences in all the cytokines analyzed (Shapiro–Wilk, *p* < 0.01) and MTT assay (Shapiro–Wilk, *p* < 0.01). Data are shown as mean ± standard deviation. The level of statistical significance was defined by a two-tailed *p* value < 0.05 throughout the study.

## 3. Results

The gas chromatographic method used to quantify thymoquinone in *N. sativa* oil was found to be linear in the range of 0.01–0.15 mg/mL, with a mean *R*^2^ value of 0.9954 (linear equation: *y* = 0.8001*x* − 0.0371) (Figure 1). The peak of thymoquinone was retained at 6.628 min, quite far away from any interference from other impurities, so apparently, no interference was found at the retention time of thymoquinone. The inter-day and intra-day precision values for the proposed method were found to be optimal (Table 1). The limit of detection (LOD) and limit of quantitation (LOQ) for thymoquinone calculated from the noise were 0.936 and 9.357 µg/mL, respectively.

The average concentrations of thymoquinone obtained were 7.2 and 4.8 mg/mL for SEO and FEO, respectively (Table 1). The low relative standard deviation (RDS) % values obtained (1.08 and 2.44%, respectively) supported the reproducibility of the method.

### 3.1. Antioxidant Assays

**Figure 2:**
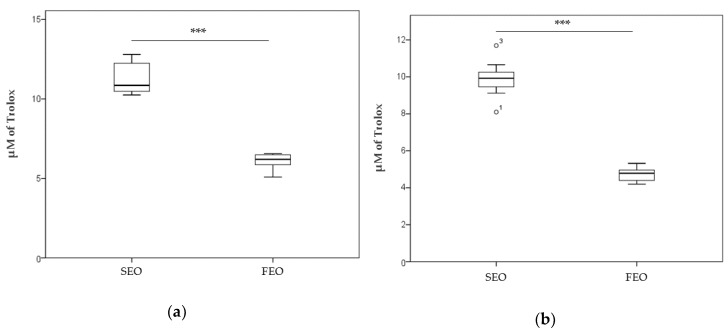
(**a**) Total antioxidant activity (TAA) measured in 2 mL of 2,2’-azinobis-(3-ethylbenz-thiazoline-6-sulfonic acid) (ABTS) solution in the presence of 20 µL of SEO and FEO. The decrease in absorbance at 734 nm of ABTS solution is referred to a standard curve obtained by using known concentrations of Trolox. (**b**) Antioxidant activity versus DPPH radical was measured in 2 mL of DPPH solution in the presence of 50 µL of SEO and FEO. The decrease in absorbance at 517 nm is referred to a standard curve obtained using known concentrations of Trolox (µM). *** *p* < 0.001. o^1^, o^3^: outliers values.

Figure 2 shows the TAA (a) and DPPH assay (b) in the SEO and FEO oils. Results from both tests highlight that SEO has a significantly higher antioxidant capacity than FEO. The µM Trolox equivalent mean values were 11.273 ± 0.935 (SEO) and 6.103 ± 0.446 (FEO) for TAA (*p* = 1.255 × 10^−7^), while mean values of 9.895 ± 0.817 (SEO) and 4.727 ± 0.324 (FEO) were obtained with the DPPH assay (*p* = 2.891 × 10^−14^).

Luminol-amplified- and lucigenin-amplified-chemiluminescence of SEO and FEO samples demonstrated that the CAT-equivalent-antioxidant capacity of SEO and FEO samples was comparable (*p* > 0.05). Similar results were obtained for SOD-equivalent-antioxidant capacity as reported in Table 2.

### 3.2. Cytotoxycity of synFEO

**Figure 3:**
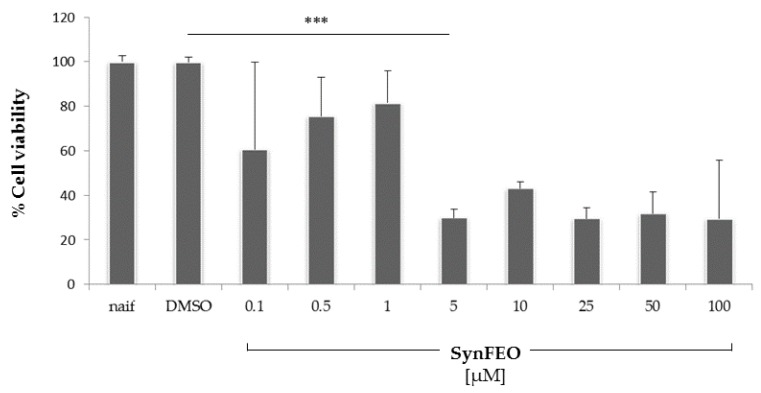
Cytotoxicity in pre-adipocyte cells after 24 h of incubation with different concentrations of synthetic thymoquinone concentration (synFEO) dissolved in dimethyl sulfoxide (DMSO). Cell viability was measured by 3-(4,5-dimethylthiazol-2-yl)-2,5-diphenyltetrazolium bromide (MTT) assay and expressed as percentage of the naif cells. 5 µM of SyntFEO resulted in a significant reduction of cell viability. Any cytotoxic effect was observed for 1 µM of SyntFEO respect to DMSO (*p* > 0.05). ***: *p* < 0.001.

Figure 3 reports the cellular viability of human pre-adipocytes incubated with different concentrations of synFEO. SEO and FEO showed similar cytotoxicity, but in both cases were lower than that of synFEO (Supplementary material Figure S1). The concentration of 1 µM was chosen for all experiments performed on cells, as this was the highest non-toxic concentration (*p* < 0.001); FEO was used on cells normalizing its volume to have a final content of thymoquinone equivalent to 1 µM. The same volume of FEO and SEO was used to screen how the impact of different thymoquinone content in the two oils can modulate the pro-inflammatory cytokine production.

### 3.3. Anti-Inflammatory Property

Pro-inflammatory cytokines (IL-1alpha, IL1beta, IL2, IL4, IL6, IL8, IL10, IL12, IL17A, IFNgamma, TNFalpha, GM-CSF) were detected in the supernatants obtained from pre-adipocytes incubated with synFEO, SEO, and FEO.

**Figure 4:**
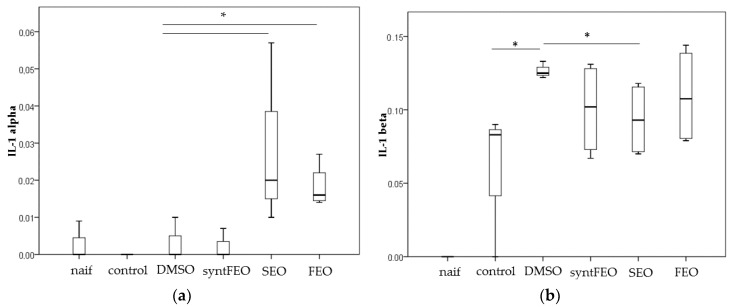

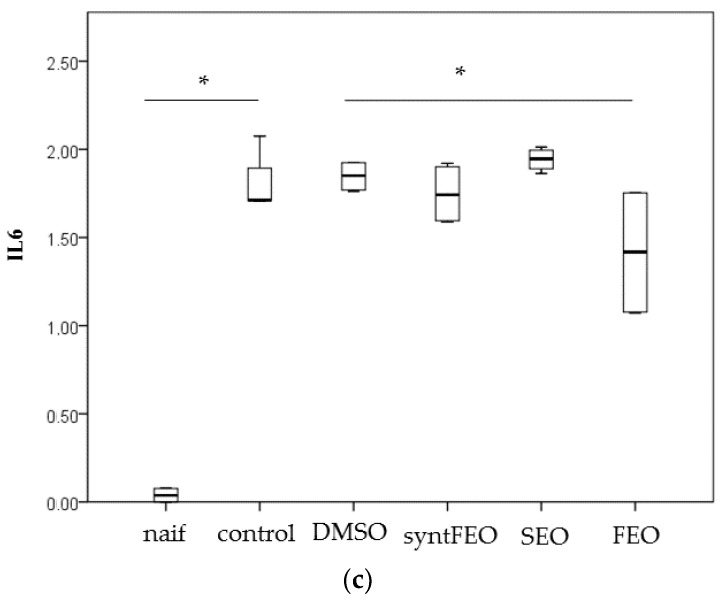
Cytokine level IL-1alpha (**a**), IL-1beta (**b**) and IL-6 (**c**) detected in the cell supernatant after incubation of cells for 24 h in the presence of 1 µM of synFEO dissolved in DMSO. FEO was diluted in DMSO and normalized to 1 µM in thymoquinone content. SEO was added to cell suspension using the same volume employed in FEO sample. * *p* < 0.05.

IL-1A was increased by both oils (DMSO vs SEO, *p* = 0.025; DMSO vs FEO, *p* = 0.018) (Figure 4a); SEO was able to decrease the level of IL1beta (*p* = 0.025) (Figure 4b), while FEO significantly reduced levels of IL6 (*p* = 0.018) (Figure 4c). SynFEO alone was not able to decrease any pro-inflammatory cytokine. No significant changes in levels of the other cytokines were observed (data not shown).

### 3.4. Antioxidant Residual Activity in the Cell Supernatant

**Figure 5:**
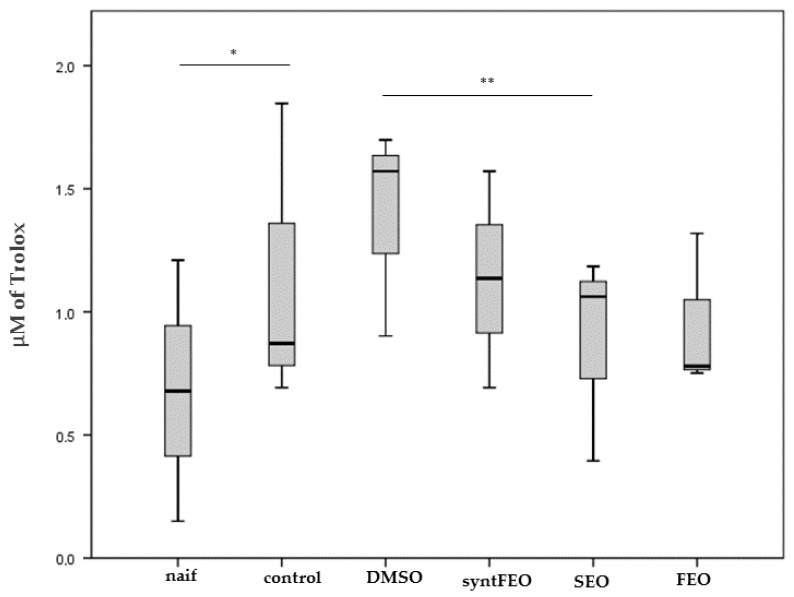
TAA measured in 2 mL of ABTS solution in the presence of 50 µL of supernatant obtained after incubation of pre-adipocytes for 24 h in the presence of 1 µM of synFEO dissolved in DMSO. FEO was diluted in DMSO and normalized to 1 µM in thymoquinone content. SEO was added to cell suspension using the same volume used for FEO sample. The decrease in absorbance at 734 nm is referred to a standard curve obtained by using known concentrations of Trolox. * *p* < 0.05 ** *p* < 0.01.

TAA measured on the pre-adipocytes supernatant shows that the antioxidant residual activity in the supernatants was significantly restored to naif level by SEO (*p* = 0.009) (Figure 5) according to in vitro results (Figure 2).

On the other hand, luminol-amplified- and lucigenin-amplified-chemiluminescence assays did not show any significant difference on the cell supernatant when the two oils were in the samples (data not shown), while DPPH assay could not be performed because this assay requires the DPPH methanolic solution, leading to supernatant precipitation.

## 4. Discussion

The present study shows that *N. sativa* oil obtained from a cultivar produced in the region Marche, located in central Italy, contains a higher thymoquinone content (7.200 mg/mL, Table 1) compared with other cultivars from the Mediterranean area, Asia, and Indonesia [8,9,20]. The level of thymoquinone is related to the fresh extraction from well-preserved seeds, while its content decreases significantly on oil storage (4.767 mg/mL, Table 1). Despite such observed relative time-dependent decrease, the content of thymoquinone in the SEO was higher than other assessments reported in the literature [8,9]. In contrast with this pattern of thymoquinone level, the antioxidant activity was higher in SEO than in FEO (Figure 2). This result is not surprising, considering that the polyphenol stability depends on room temperature, light, time of storage, and humidity [21,22]. Prolonged storage promotes both chemical and enzymatic oxidation of polyphenols and of other macromolecules; however, antioxidant activity is maintained in well-preserved oil. Furthermore, for an optimal preservation, cryostorage techniques have been suggested, because each seed has a tolerance of desiccation and longevity in their storage [23]. The enzymatic activity in the seeds can be modified because its carbonyl and amino groups can also undergo oxidation. Phenolic compounds can be transformed into quinones through polyphenol oxidases, which utilize oxygen to catalyze hydroxylation and dehydrogenation of phenolic compounds [24]. Although *N. sativa* seeds were stored at a controlled temperature (14 °C) in a dark room, the time of their storage was about 6 months, and this might have been responsible for the natural decay of seed antioxidant properties, which were maximum in the fresh seeds and in its extracted oil (SEO) that was properly conserved. Moreover, the key role of all components of oils can be observed in the TAA and DPPH assays performed on the two oils (Figure 2), whereby the same volume of SEO, containing lower thymoquinone amounts than FEO, exerts a higher antioxidant capacity; these data indicate that the oil components exert a synergistic antioxidant activity. Finally, observing the TAA on antioxidant residual activity of cell supernatants, SEO appears to have a higher residual activity, which may be associated with its own elevated antioxidant capacity in comparison with FEO (Figure 5).

Here, we have used the SGBS pre-adipocytes strain to screen anti-inflammatory properties of oils, because these cells are an in vitro model of human subcutaneous pre-adipocytes [25,26,27]. SGBS cells have been validated as an in vitro model for obesity and cancer research in humans [28]. Release of pro–inflammatory cytokines in hypertrophied adipocytes and adipose tissue–resident immune cells has been associated with low-grade systemic inflammation that can contribute to develop metabolic inflammation, which is itself a starting point of several diseases, such as cancer, neurodegeneration, diabetes, etc. [29,30]. Plasma IL-1beta and IL-6 can trigger pancreatic islet dysfunction in animals [31]; IL-1beta can perturb the insulin-signaling pathway, calcium storage and stress responses [31]. Metabolic inflammation in obese individuals has been associated with the release of pro-inflammatory cytokines such as TNF-*α*, IL-6, from adipose tissue, while the adipose tissue in lean subjects produces anti-inflammatory cytokines (e.g., transforming growth factor beta, interleukin (IL)-10, IL-4, IL-13, IL-1 receptor antagonist (IL-1Ra), and apelin) [27]. IL-6 was increased in human obese individuals and in plasma of type 2 diabetic patients [27], because one-third of its production occurs in the white adipose tissue, mainly by macrophages and partially through adipocytes [32]. The IL-6 level increases with the free fatty acid level, body mass index, and waist circumference increase. Besides, this interleukin can stimulate fatty acid metabolism dysregulation, and its chronic increase in the adipose tissue and the liver has been associated with insulin resistance and the release of adiponectin, influencing adipocyte differentiation [27]. Our data on stimulated SGBS pre-adipocytes show that IL-6 release was significantly decreased by FEO (Figure 4c). These data are particularly relevant considering the systemic role of this interleukin and its negative impact on health; in particular, the risk of developing type 2 diabetes is increased when there is the combination of increased IL-6 and IL-1beta [33]. Both interleukins are significantly increased by pro-inflammatory treatment of cells, and the capacity of FEO to decrease IL-6 underlines the key protective function of this oil. Furthermore, SEO was able to significantly decrease IL-1beta (Figure 4b), which works synergistically with IL-6. The protective modulation of FEO and SEO on IL-6 and IL-1beta, respectively, was not observed when purified thymoquinone was used at the same concentration in FEO on the SGBS pre-adipocytes cultures. These data suggest that thymoquinone alone, at the concentrations used in this study, is not able to control IL-6 release, but its high content, together with the other components of *N. sativa* oil, works synergistically to control the release of this interleukin. Furthermore, IL-1beta is modulated more by the other components of *N. sativa* oil than by thymoquinone alone. Conversely, both oils increase IL-1alpha levels (Figure 4a); IL-1alpha and IL-1beta, which both bind to IL-1R, and seem to induce the same biological effects; they stimulate inflammation-promoting activation of NF-kappaB by Myeloid differentiation primary-response gene 88 (Myd88) activation, which leads to pro-inflammatory gene expression. However, the two interleukins seem to have different response times; IL-1apha accumulates in the initial phase of inflammation and can recruit neutrophils, while IL-1beta later recruits macrophages [34]. IL-1alpha seems to have two roles: in the nucleus of cells it has regulatory functions, while, when released, it promotes inflammation [34]. The differences between the two IL-1 forms might suggest that IL-1alpha mediates the primary signal of inflammation due to tissue injury, likely because of paracrine actions. Moreover, IL-1alpha is present under homeostatic conditions at the level of the nucleus, cytosol, and membrane, and it increases with inflammation, while IL-1beta is not present under homeostatic conditions [35]. According to its role, IL-1alpha, in our experimental conditions, increases easily in the SGBS pre-adipocytes medium after incubation with the oils, underlining how this interleukin is sensitive to changes in cellular homeostasis.

Further studies will now be addressed to screen the in vivo influence of these oils in the control of inflammatory and anti-oxidant responses in a low-grade inflammation animal model.

## 5. Conclusions

In conclusion, the *N. sativa* oil produced in the Marche region, located in central Italy, has a high level of thymoquinone and a marked antioxidant activity. Data from the same seeds show that thymoquinone can be preserved better in well-stored seeds (controlled temperature (14° C) in a dark room), while anti-oxidant properties are more stable in well-stored oil (controlled temperature (18–20° C) in a dark room). As the oil extraction method is the same used for FEO and SEO, the differences observed can be associated with the stability of molecules in seeds or oil. FEO, containing more thymoquinone, significantly reduces IL-6 levels, while SEO inhibits IL-1beta and has higher anti-oxidant activity. Proper biological responses can be measured in oils, but not in purified thymoquinone. The extraction of the oil from fresh and old seeds might be chosen according to its final use.

## Figures and Tables

**Table 1:** Thymoquinone concentrations (mg/mL) in stored extracted oil (SEO) and fresh extracted oil (FEO). Intra Day (a) and Inter Day (b) reproducibility.

**(a) Intra Day**		
**Sample**	**Mean ± SD** **mg/mL**	**RDS%**
SEO	4.767 ± 0.116	2.44
FEO	7.200* ± 0.078	1.08
**(b) Inter Day**		
**Sample**	**Mean ± SD** **mg/mL**	**RDS%**
SEO	4.834 ± 0.117	2.43
FEO	7.118* ± 0.083	1.18

* *p* < 0.0001 vs sample SEO; RDS: relative standard deviation; SD: standard deviation.

**Table 2:** Luminol-amplified- and lucigenin-amplified chemiluminescence (CL) of SEO and FEO. Antioxidant activity toward H_2_O_2_ and O_2_^−.^ produced in the Luminol-amplified- and lucigenin-amplified-system respectively, was quantified using known amounts of superoxide dismutase (SOD) and catalase (CAT) enzymes. *p* > 0.05.

Sample (10 µL)	Luminol-Amplified CL (Scavenger Activity H_2_O_2_)	Lucigenin-Amplified CL (Scavenger Activity to O_2_^−.^)
SEO	140.68 ^1^ ± 4.66	0.0138 ^2^ ± 1.54 × 10^−5^
FEO	132.78 ^1^ ± 11.33 ^1^	0.0137 ^2^ ± 4.175 × 10^4^

**^1^** Corresponding to µg CAT in sample. **^2^** Corresponding to µg SOD in sample.

## Supplementary Materials

The following are available online at https://www.mdpi.com/2076-3921/8/2/51/s1. **Figure S1.** Cytotoxicity in pre-adipocyte cells after 24 h of incubation with different concentrations of SEO, FEO and synFEO solved in DMSO. Cell viability was measured by MTT assay and expressed as percentage of the naif cells.
