# Supplementary figures and images for: Antioxidant and Anti-Inflammatory Properties of Nigella sativa Oil in Human Pre-Adipocytes
Source: Antioxidants (Basel). 2019 Feb 25;8(2):51. doi: 10.3390/antiox8020051 (PMC6406245; doi:10.3390/antiox8020051)

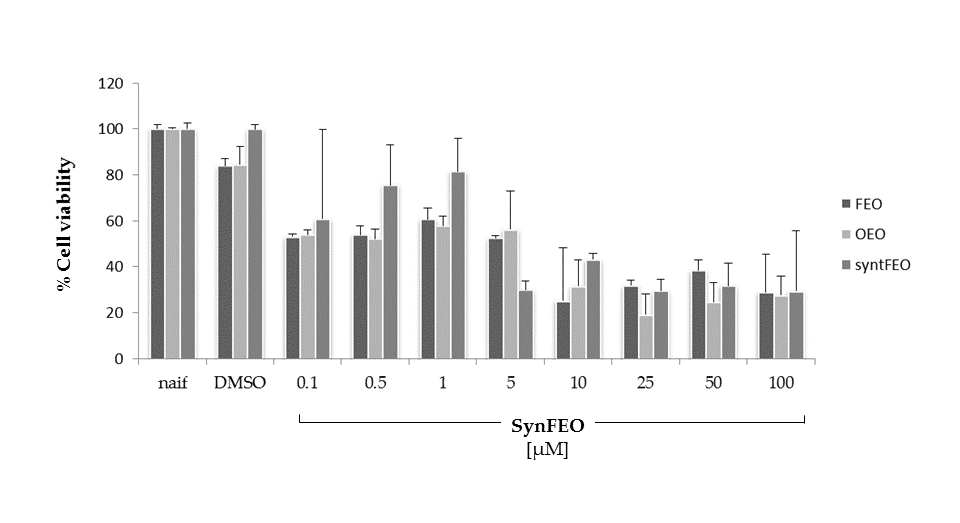

Supplement: Supplementary file 1 [file antioxidants-08-00051-s001.png]
